# Total laparoscopic pancreaticoduodenectomy with left posterior superior mesenteric artery first-approach and plexus-preserving circumferential lymphadenectomy: step-by-step technique with a surgical case report (with video)

**DOI:** 10.1186/s12957-022-02730-y

**Published:** 2022-08-27

**Authors:** Thanh Khiem, Ham Hoi, Tuan Hiep, Kim Khue, Van Duy, Yosuke Inoue, Hong Son, Duc Dung

**Affiliations:** 1 Department of Gastrointestinal and Hepato-pancreato-biliary Surgery, Bach Mai Hospital, Hanoi, Vietnam; 2 Department of Surgery, Hanoi Medical University, 1st Ton That Tung Street, Dong Da, Ha Noi, 11521 Vietnam; 3 Department of Hepato-Biliary-Pancreatic Surgery, Cancer Institute Hospital, Japanese Foundation for Cancer Research, 3-8-31 Ariake, Koto-ku, Tokyo, 135-8550 Japan; 4 Department of Oncology, Viet Duc University Hospital, Hanoi, Vietnam; 5 Department of Surgery, Thai Binh Medical University, Thai Binh, Vietnam

**Keywords:** Total laparoscopic pancreaticoduodenectomy, Left posterior SMA first-approach, SMA plexus-preserving and circumferential lymphadenectomy

## Abstract

**Introduction:**

Total laparoscopic pancreaticoduodenectomy (tLPD) for cancer of the Vater remains a challenging procedure. Recently, several meta-analyses showed the superior aspects of “superior mesenteric artery (SMA)-first approach,” “systematic mesopancreas dissection,” and “circumferential lymphadenectomy around SMA” in increasing R0 resection rate and reducing postoperative complications including pancreatic fistula and bleeding as well as improving overall survival particularly.

**Case presentation:**

Our patient is a 70-year-old female with a no special medical history, recruited because of jaundice. She was referred for pancreaticoduodenectomy because of a 10-mm-sized mass in distal bile duct referred to as Vater’s tumor. We used 5 trocars, and the patient was placed in a Trendelenburg position. The transverse colon was lifted, the first loop of the jejunum was pulled to the left, and lymph node groups 14th and 15th were removed en bloc and then exposed the SMA from the anterior to the left posterior side from the caudal side to the origin. The first jejunal vessels and the posterior inferior pancreaticoduodenal artery were ligated as well as the extensive mobility of the duodenum and head of the pancreas from the left side. The systematic mesopancreas dissection from the right site of the SMA will be easily and conveniently done afterwards. Histopathological examination of ypT2N1 indicated that 1 of the 22 lymph nodes was positive, which was 1 of 7 LN no. 14. Pathological results showed a Vater adenocarcinoma with all margins being negative.

**Conclusions:**

This technique was safe and effective to perform precise level 2 mesopancreas dissection and complete lymphadenectomy around SMA without dissection of pl-SMA in laparoscopic field.

**Supplementary Information:**

The online version contains supplementary material available at 10.1186/s12957-022-02730-y.

## Introduction

Pancreatic head and periampullary tumors, especially pancreatic cancer, are still aggressive lethal diseases, being set to become the second most common cause of cancer-related mortality within the next few years [1]. Pancreaticoduodenectomy (PD) remains the only curative surgical option for pancreatic head and periampullary tumor. In combination with neoadjuvant chemotherapy, the indication of PD is not only in the resectable group but also extend to the group of locally advanced tumors, with up to 60% of patients with previously unresectable disease becoming candidates for curative surgery [2]. The development of technology and minimally invasive tendency in medicine and minimally invasive pancreaticoduodenectomy, especially total laparoscopic pancreaticoduodenectomy (tLPD), is not out of this way. Recent systematic reviews showed that LPD was superior against open PD in aspects of inoperative blood transfusion, wound and pulmonary infection, and shorter hospital stays, and there are no differences of relevant postoperative pancreatic fistula (POPF), severe complications, postoperative mortality, retrieved lymph nodes (LNs), and R0 resection rate [3, 4]. However, to achieve the safety and effectiveness, they also recommended that LPD should be done by surgeons with expertise and through learning curve in high-volume centers [3, 4].

With up-to-date knowledge about pathological aspects of oncological process and involvement, there are several new techniques or approaches to make PD becoming complete as an oncologic surgery. And with most discussions and improvements, they are the SMA-first approach, circumferential lymphadenectomy around SMA with preservation of pl-SMA, and systematic mesopancreas dissection. Many reports have discussed and shown the superior aspects of these techniques against convenient approach in open LD, but there were few reports taken in tLPD. So, herein, we reported a technique of tLPD that combined left posterior SMA-first approach and SMA LN circumferential as well as systematic mesopancreas (MP) dissection with pl-SMA preserving.

## Case presentation

This is a reported case of a 71-year-old female patient without any medical history. She admitted to the hospital because of progressive jaundice, and it lasted 1 month but no complaint of abdominal discomfort. Her past medical history was unremarkable. There were no significant findings on physical examination with the exception of severe malnutrition. Height was 160 cm, and weight was 50 kg. Laboratory findings were as follows: total bilirubin, direct bilirubin, and albumin were 233.0, 128.0 μmol/l, and 3.25 g/dL, respectively, and amylase was within the normal range. The serum level of carbohydrate antigen (CA) 19-9 was 20.2 U/mL, and carcinoembryonic antigen (CEA) was 3.02 ng/ml. Abdominal contrast-enhanced computed tomography (CT) scan revealed a 10-mm solid hypovascular mass in the periampullary region and distal common bile duct as regards the ampullary tumor. The common bile duct and the main pancreatic duct on the distal side of the mass were dilated at 20 mm and 9 mm in diameter, respectively. The endoscopic ultrasound (EUS) revealed that a 1.2 × 1.4 cm hyperechoic mass in the ampulla of Vater. There was no evidence of lymph node metastasis, peritoneal dissemination, or distant organ metastasis. The upper endoscopy revealed a mass in the duodenal papilla of Vater, and the biopsy result was primary duodenal papilla adenocarcinoma. The final diagnosis was an adenocarcinoma tumor of the ampulla of Vater with TNM staging which was cT1N0Mx according to the American Joint Committee on Cancer (AJCC) 8th Staging [5]. MDT meeting consisted of surgeons, physicians, clinical and medical oncologists, radiologists, pathologists, and clinical nurse specialists (CNSs) which were organized to make clinical decisions. The informed consent was signed, and tLPD following the described technique was performed. The operation time was 480 min, and the estimated blood loss was 50 ml. With no postoperative complications as well as no diarrhea, the patient was discharged on POD8 uneventfully. The pathological result is as follows: intestinal-type ampulla of Vater carcinoma; metastasis to 01/29 lymph node metastasis (LN group 14) was confirmed. The resection margin was negative of tumor involvement (R0).

### Surgical procedure

Trocars’ placement. We used 5 trocars: one 10 mm trocar was placed through the umbilicus for camera; two 12 mm trocars were placed at the midclavicular line 1 cm higher compared to the umbilicus in the right and left sides for the instrument; two 5 mm trocars were placed at the right and left subcostal. The surgeon stands on the right side of patient in the SMA’s dissection phase and changes to the middle position when dissecting the posterior surface of the artery with the rest of the surgical phases, with the second and third assistants holding the middle and right cameras and the first assistant standing on the side left.

Step 1: We mobilized the first jejunal loop and approached the superior mesenteric artery (SMA) from the left posterior side. After exploring the peritoneum to exclude metastases, the right-sided assistant lifts the mesentery of transverse colon upward, and the left-sided assistant pulls the first jejunum to the left side. We open the peritoneal along the meso of the first jejunal loop, dissecting the lymph node group 14 and group 15 along the first jejunal artery (FJA), middle colic artery, and right colic artery. We proceeded to exposing the FJA adjacent to the superior mesenteric artery (SMA) and isolated and ligated close to the origin of the FJA. Dissection of the left border of the origin of SMA just above the left renal vein (LRV) including LN station no. 14 or left-sided SMA LNs was done (Video 1).

After transecting the first jejunal loop with GI stapler with the corresponding mesenteric, the ligament of Treitz was then mobilized with dissection of the 3rd and 4th portions of the duodenum. We assessed and classified the anatomy of the first jejunal vein (FJV) before and during surgery. In the case of the anterior, a simple approach and dissection should be done. In most of the cases, we found the common trunk between the FJV, and the inferior pancreaticoduodenal vein (IPDV) goes posteriorly to the SMA: after ligation of the FJA, the SMA was dissected and lifted, the FJV was approached and hemo-locked just below the left side of the SMA, and then, we continued in dissecting the first jejunal loop and the pancreatic uncinate process behind this vein.

**Fig. 1:**
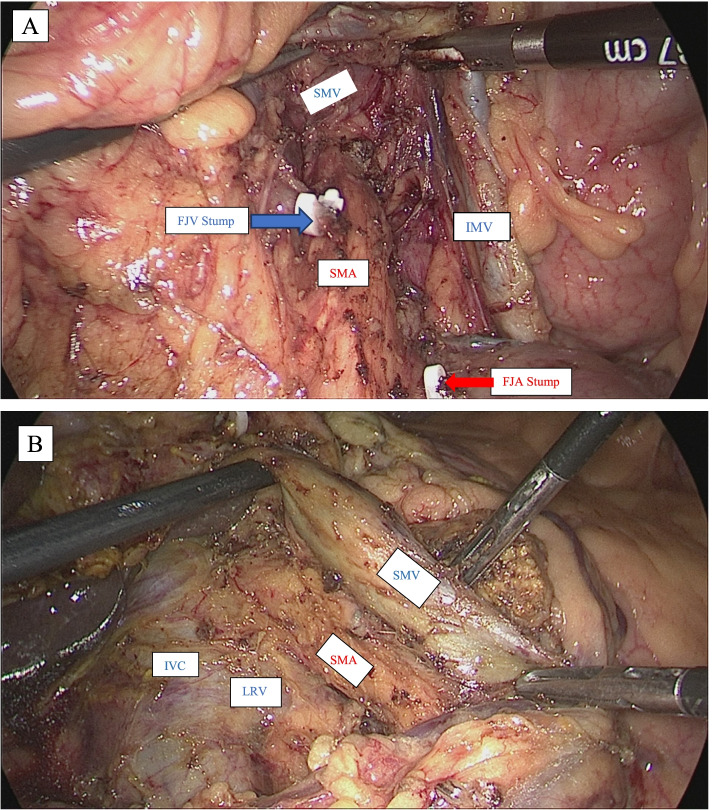
Preservation of the neural plexus around the SMA (**A** left side; **B** right side) (SMA, superior mesenteric artery; SMV, superior mesenteric vein; IMV, inferior mesenteric vein; PV, portal vein; IVC, inferior vena cava; FJA, first jejunum artery; FJV, first jejunum vein; LRV, left renal vein)

The main surgeon changes to middle position. We continued dissecting on the posterior side of the SMA and ligated the posterior pancreaticoduodenal artery (PPDA) (if any) on this posterior surface. We proceeded to dissecting the superior border and then passing the superior border to the right border to separate the SMA from the SMV. The neural plexus around the SMA is carefully preserved (Fig. 1). We found that extensive mobilization of the duodenum and head of the pancreas from the left side (Kocher maneuver) to the proximal right peritoneal fold of the duodenum is easy during this phase.

Step 2: We have done mobilization of the entire pancreaticoduodenal mass and approached the superior mesenteric vein from the posterior side of the pancreas. We detached the great omentum from the transverse colon and detached the hepatic flexure from the duodenum and pancreatic head to the lower border of the pancreas. We proceeded to exposing the right margin of the superior mesenteric vein (SMV) and dissected the gastrocolic trunk of Henle (the confluence of the veins of the pancreatic head and the right colon before flow into the SMV). Mobilizing the hepatic flexure of the colon and the duodenum was done. We then exposed the posterior surface of the duodenum and the head of the pancreas to the left border of the aorta above the renal vein at the origin of the SMA.

Step 3: We have transected the stomach and pancreatic neck and dissected the hepatoduodenal ligament and the celiac lymph nodes. We started dissecting the right gastroepiploic artery (RGEA), right gastric artery from the level of the antrum. The stomach was transected using a stapler about 2 cm from the pylorus. The right gastric artery (RGA) was transected at its origin; then, we dissected the lymph node groups 8a, 12a, 7, and 9 in front of the hepatoduodenal ligament to the origin of the left gastric artery and superior border of the celiac trunk. The gastroduodenal artery (GDA) is dissected and ligated, and isolating the common hepatic artery (CHA) from the origin to the GDA’s bifurcation was done. The tissue plane between the inferior surfaces of the pancreatic neck is then blunt dissected, and we revealed the anterior surface of the SMV and the portal vein (PV). A nelaton was inserted from the inferior to the superior side of the pancreas. We transected the pancreas at the pancreas neck and exposed the pancreatic duct with at least 5 mm in length. After resection of the pancreatic neck, we carried on dissecting lymph node groups 8b and 12p in the inferior surface of the PV and right-sided celiac artery (CA).

Step 4: We isolated the entire pancreaticoduodenal mass from the SMA, SMV, and PV. The proximal loop of the jejunum is passed to the right side through retroperitoneal defect. We systematically performed dissecting the meso-pancreas from SMV, SMA, and PV. We resected the entire mesopancreas on both anterior and posterior sides of the SMA including the right-sided SMA LNs (Video 2). This procedure is often quite difficult with the convenient technique but becomes much easier with left posterior SMA-first approach. We continued dissecting the lymph nodes posterior to the main bile duct up to the proximal side of hepatic hilum. This step was done easily with our technique after the entire pancreaticoduodenal mass was mobilized subtotally after this step. After the cholecystectomy, the common bile duct was dissected and divided at the level of the right hepatic artery. Specimens were taken en bloc: duodenal and pancreatic head, lymph node groups 5, 6, 7, 8, 9, 12a, 12b1, 12b2, 12v, 13, 14p, and 14d (or left-sided SMA LNs), and the right-sided SMA LNs and 15.

Step 5: Small opening at umbilicus, remove the sample.

Step 6: Reconstruction phase. The first loop of the jejunum is then brought through the transverse mesocolon. Pancreatojejunostomy is performed with double-layer end-to-side, modified Blumgart fashion. The inner layer is duct to mucosa anastomosis, using 5-0 monosyl suture (6 stitches: 3 posterior, 3 anterior). We placed an internal drainage with a plastic stent. The second layer is 2 interrupted U-shape suturing between the posterior and anterior pancreatic walls and seromuscular layer of the jejunum using Prolene 3/0. Hepaticojejunostomy is performed distal to the pancreato-jejunostomy about 10 cm with one-layer fashion. The anastomosis of the common hepatic duct to the jejunum in an end-to-side fashion using 5-0 monosyl continuous suture for the posterior layer and interrupted suture for the anterior layer was done. A distal loop of jejunum approximately 60 cm distal to defect in the transverse mesocolon is brought antecolic. Antecolic gastro-jejunostomy performed via the enterotomy and gastrotomy using a GI stapler. Defect in gastrojejunostomy is oversewn with interrupted 3-0 Vicryl suture (Video 3).

## Discussion and conclusion

The history of the concept artery first approach begun in 1993, when Nakao et al. first represented a technique of “isolated pancreatectomy using catheter bypass of the portal vein” for pancreatic head carcinoma, while all arteries and drainage veins that supply the pancreatic head region are ligated and divided [6]. After that, the concept “SMA-first approach” was firstly introduced by Patrick Pessaux et al. in a technical article in 2006 [7]. This technique started from some reasons: firstly is the requirement of accurate preoperative stage and resectability of the tumor following guidelines of the National Comprehensive Cancer Network (NCCN) [8], and secondly is the high rate of postoperative morbidity and mortality strongly related to the complexity of anatomical variation of superior mesenteric vessel branches and tributaries. In concept, the term “SMA-first approach” means exploration of superior mesenteric vessels as well as celiac trunk and portal vein to detect the state of tumoral invasion of these vessels and determine the resectable conditions before the point of no return (the step of pancreatic neck’s dissection or bile duct division) [7, 9]. Until now, many methods have been developed to achieve the “SMA-first approach” concept in open PD, and according to our knowledge, there are totally six methods that have been summarized by Sanjay, P et al. with advantages, disadvantages, and indications of each approaches [10]. Recently, several meta-analyses showed the superior aspects of “SMA-first approach” in R0 resection rate and postoperative complications including pancreatic fistula and bleeding as well as overall survival [11, 12]. In a recent systematic review by Nagakawa Y. et al, with laparoscopic PD, methods for the “SMA-first approach” concept were modified based on these approaches in open PD and categorized into four approaches included: anterior approach, posterior approach, right approach, and left approach [13]. In posterior approach, the posterior side of the SMA was exposed above the LRV from the right side [14] or from the caudal side [15]. In left approach, Cho et al. described a surgical technique to divide the common artery of the IPDA and the FJA by pulling the proximal jejunum to the right to visualize the ligament of Treitz and the origin of the SMA is visualized just above the LRV [16]. In our technique, we divide the FJA or the common artery of the IPDA and the FJA after removal of lymph node group 15 and circumferential SMA lymph nodes on the left anterior side to the posterior side by pulling the proximal jejunum to the left; then, after dissecting the first jejunal loop, the SMA was exposed from the left and caudal side. So, we call our technique “the left posterior first approach.”

**Fig. 2:**
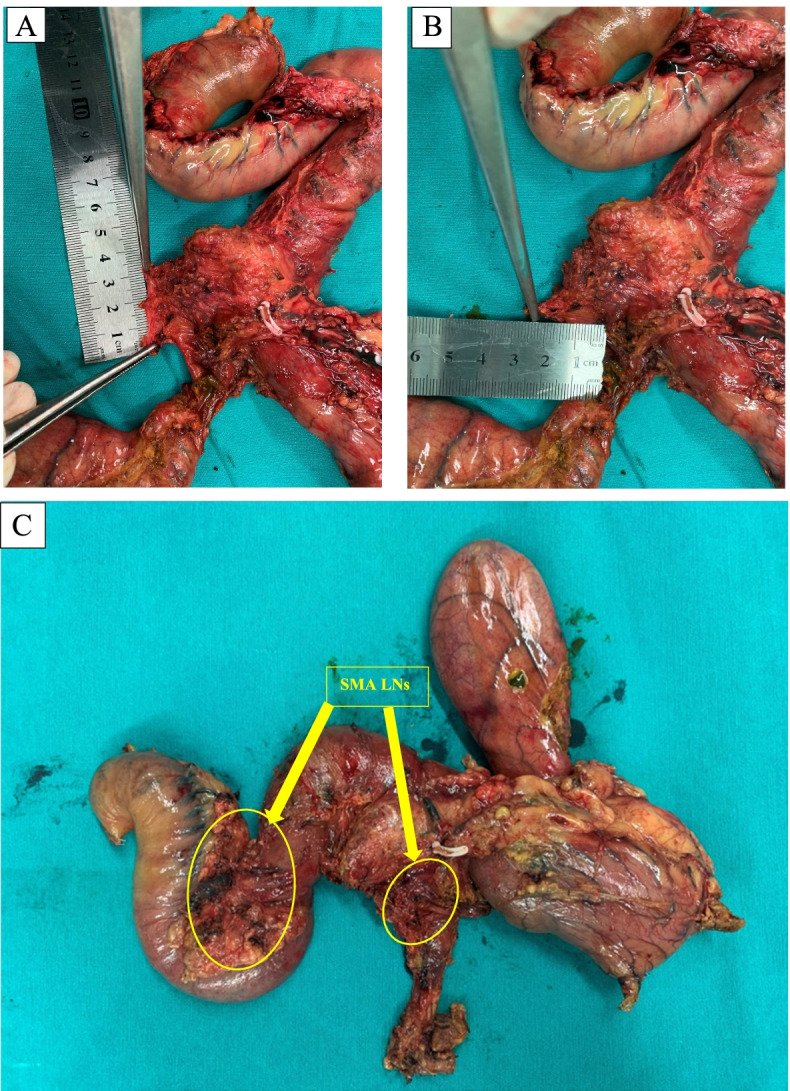
Specimens. **A**, **B** The mesopancreas size was recorded systematically in our protocol. **C** SMA LNs including right-side and left-side en bloc

Besides “SMA-first approach,” total or systematic mesopancreas (MP) dissection is also a concept that has been paid a lot of attention recently [17]. Started by Gockel et al. in 200 7[18], in definition, mesopancreas was a retropancreatic well-vascularized and nerve-rich structure surrounding the SMA, including the first and second nerve plexuses of the pancreas head (plPh-I and plPh-II) according to the new classification of Japan Pancreas Society as well as some landmarks: inferior pancreaticoduodenal arteries (IPDAs), jejunal arteries (JAs), jejunal veins (JVs); and lymph nodes (LNs) [19]. In anatomical aspect, the mesopancreas connects the pancreatic head to the SMA and right celiac ganglion [17]. Many retrospective studies were done and summarized in a systematic review by J. M. Ramia et al. in 2018 with conclusions of MP excision increased the R0 resection rate, so that improved the oncological outcomes [20]. According to Inoue et al., there are 3 levels of the systematic dissection of mesopancreas, which were applied due to type and extent of pancreatic head tumors, and level-2 systematic MP dissection includes en bloc LNs dissection in the MP by the central vessel ligation technique, which is implemented to ampullary/lower bile duct/duodenal cancer or selective invasive pancreatic ductal cancer [17]. In our technique, the mesopancreas was resected systematically en bloc with entire pancreaticoduodenal mass. The mesopancreas size was recorded in this case and all other PD cases we have done (Fig. 2).

One of the most impact factors that influence the survival outcomes of resectable pancreatic ductal adenocarcinoma (PDAC) is high frequency of lymph node metastasis, especially the left side of LNs around SMA, due to right-sided soft tissues including LNs usually resected in conventional PD [21, 22]. Following the new Japanese LN station system, the LN no. 14 is divided to LN no.14p and 14d, located in the left side of the SMA (left-side LNs of SMA), and located in an anatomical–surgical layer of the “meso-pancreatoduodenum,” which was along the IPDA and the FJA [23, 24]. So, systematic MP dissection does not include left-side SMA LNs. Otherwise, the rate of left LNs no.14 metastasis according to Okada’s study was 12% [25]. There were some preliminary evidences that proved the superior survival outcomes of SMA LN circumferential dissection and not increased the rate of postoperative complications especially postoperative diarrhea [21]. And in another aspect, the concept of right-half dissection of the SMA nerve plexus along with SMA LNs circumferential dissection, in order to accomplish R0 resection with the potential for nerve plexus invasion, showed no significant difference in oncological outcome, as well as significantly increased the rate of postoperative diarrhea requiring opioids according to recent studies and trials [26, 27].

## Conclusion

Herein, we reported a successfully first-described total laparoscopic left posterior first-approach and superior mesenteric artery plexus-preserving pancreaticoduodenectomy with circumferential dissection of lymph nodes in a patient of primary duodenal papilla adenocarcinoma with staging pT2N1M0 with negative margin (R0 resection). There were no short-term complications. We think this technique was safe and effective to perform precise level 2 dissection in laparoscopic field. Further investigations and follow-up must be done to evaluate the long-term outcomes of our technique.

## Supplementary Information

**Additional file 1: Video 1.** Dissection the left border of the origin of SMA just above the left renal vein (LRV), including LN station no. 14 or left side of SMA LNs.

**Additional file 2: Video 2.** Resection of the entire mesopancreas both anterior and posterior sides of SMA including right-side of SMA LNs.

**Additional file 3: Video 3.** The reconstruction phase.

## Data Availability

Data is available upon reasonable request and with permission of Bach Mai Hospital.

## Abbreviations

AJCC: American Joint Committee on Cancer
CA: Celiac artery
CEA: Carcinoembryonic antigen
CHA: Common hepatic artery
CT: Computed tomography
EUS: Endoscopic ultrasound
FJA: First jejunal artery
FJV: First jejunal vein
GDA: Gastroduodenal artery
GI: Gastrointestinal
IPDA: Inferior pancreaticoduodenal artery
IPDV: Inferior pancreaticoduodenal vein
LN: Lymph nodes
LRV: Left renal vein
NCCN: National Comprehensive Cancer Network
PDAC: Pancreatic ductal adenocarcinoma
POPF: Postoperative pancreatic fistula
PL: Neural plexus
PPDA: Posterior pancreaticoduodenal artery
LPD: Laparoscopic pancreaticoduodenectomy
SMA: Superior mesenteric artery
SMV: Superior mesenteric vein
RGA: Right gastric artery
